# Tuberculosis disease burden in China, Japan, and South Korea: A population-based observational study of trends from 1990 to 2023 and predictions for 2035

**DOI:** 10.1097/MD.0000000000049112

**Published:** 2026-05-29

**Authors:** Huan Yao, Yujie Qian, Zhi Xu

**Affiliations:** a Department of Respiratory and Critical Care Medicine, Zhangjiagang Fifth People’s Hospital, Zhangjiagang, Jiangsu, China; b Science and Education Department, Zhangjiagang Fifth People’s Hospital, Zhangjiagang, Jiangsu, China.

**Keywords:** attributable risk factors, Bayesian age-period-cohort model, estimated annual percentage change, Global Burden of Disease, tuberculosis

## Abstract

Tuberculosis (TB) remains a leading infectious killer worldwide, with the World Health Organization End TB Strategy targeting 90% and 95% reductions in incidence and mortality by 2035. Long-term comparative analyses across China, Japan, and South Korea remain lacking. This study aimed to systematically analyze trends in the TB disease burden in China, Japan, and South Korea from 1990 to 2023 and project future burden trajectories through 2035. Using the Global Burden of Disease 2023 database, data on TB incidence, prevalence, mortality, and disability-adjusted life years (DALYs) were extracted. The estimated annual percentage change was used to evaluate long-term trends in the age-standardized incidence rate (ASIR), age-standardized prevalence rate (ASPR), age-standardized mortality rate (ASMR), and age-standardized DALYs rate. The population attributable fraction was calculated to quantify the contributions of smoking, high alcohol use, and high fasting plasma glucose. The Bayesian Age-Period-Cohort model was applied to project disease burden from 2023 to 2035. From 1990 to 2023, ASIR, ASMR, and age-standardized DALYs rate declined continuously in all 3 countries, exceeding the global average of decline. By 2023, China had the highest ASIR (38.43 per 1,00,000) and ASPR (30,714.91 per 1,00,000), while Japan recorded the lowest ASIR (3.94 per 1,00,000); both Japan and South Korea fell below the global ASPR. China showed the greatest mortality reduction (estimated annual percentage change = −8.61), with all 3 countries’ ASMR well below the global level (11.64 per 1,00,000). TB burden increased with age, with males consistently bearing a greater burden than females. While smoking remained the leading attributable risk factor, high fasting plasma glucose rose continuously across all 3 countries and surpassed smoking in South Korea as the primary contributor to TB deaths and DALYs. Bayesian age-period-cohort projections indicated a continued overall decline through 2035, except for a transient rise in China’s ASPR before stabilization. The TB burden has declined steadily in China, Japan, and South Korea, though at different paces. Population aging and rising metabolic risk factors remain key challenges. These findings support region-specific strategies – integrated diabetes–TB management, latent TB screening in older adults, and sustained tobacco control – to advance the World Health Organization End TB 2035 targets in East Asia.

## 1. Introduction

Tuberculosis (TB), caused by Mycobacterium tuberculosis, remains one of the leading infectious causes of death worldwide.^[1]^ Recent estimates from the 2022 global burden review indicate that approximately 10.6 million people (133 per 1,00,000) developed TB in 2022 (a 2.9% increase from 2021), and about 1.3 million died from the disease (a 6.4% decrease from 2021).^[2]^ The World Health Organization End TB Strategy set ambitious targets of 95% and 90% reductions in TB mortality and incidence, respectively, by 2035 compared with 2015 levels.^[3]^ However, achieving these goals remains a formidable challenge globally.

China, Japan, and South Korea are representative countries in East Asia that differ markedly in economic development, demographic structure, and TB control policies, resulting in distinct trajectories of disease burden. China remains one of the countries with the highest TB burden worldwide; despite a sustained decline in incidence rates, the sheer size of its population keeps the absolute number of cases persistently high.^[4]^ Japan has reduced its TB incidence to relatively low levels, yet rapid population aging and the associated decline in immune function continue to pose new challenges for TB control.^[5]^ South Korea has achieved substantial reductions in disease burden through enhanced active case-finding and standardized treatment, although the infection risk among elderly populations remains a concern.^[6]^ Previous studies have identified smoking, high fasting plasma glucose, and high alcohol use as major attributable risk factors for TB morbidity and mortality.^[7,8]^ With the rising prevalence of diabetes across East Asia, the attributable contribution of hyperglycemia to TB burden has grown increasingly prominent, gradually reshaping the traditional risk factor landscape previously dominated by smoking.^[9]^ Furthermore, the TB burden is unevenly distributed across age and sex groups, with older adults and males consistently bearing a disproportionately greater burden.^[10]^

The Global Burden of Disease (GBD) study, led by the Institute for Health Metrics and Evaluation, provides standardized estimates of disease burden across 204 countries and territories and represents one of the most authoritative and comprehensive global health data resources currently available.^[11]^ Nevertheless, a systematic comparative analysis of TB disease burden across China, Japan, and South Korea with long-term trend projections based on GBD data is still lacking. Therefore, this study draws on the GBD 2023 database to systematically analyze trends in TB incidence, prevalence, mortality, and disability-adjusted life years (DALYs) across the 3 countries from 1990 to 2023, examine age- and sex-specific disparities and the dynamic changes in major attributable risk factors, and project disease burden trajectories through 2035 using the Bayesian age-period-cohort (BAPC) model, with the aim of providing evidence-based support for regional TB control policy.

## 2. Materials and methods

### 2.1. Data sources

Data for this study were obtained from the GBD 2023 database, developed by the Institute for Health Metrics and Evaluation at the University of Washington. This database encompasses data on disease incidence, prevalence, mortality, and DALYs across 204 countries and territories from 1990 to 2023, representing one of the largest and most comprehensive global health data resources currently available. It has been widely applied in global public health policy development and health research.^[12]^ As this study involved only secondary analysis of publicly available, de-identified data from the GBD 2023 database, formal ethical approval from an institutional review board was not required. All procedures were conducted in accordance with the Declaration of Helsinki.

### 2.2. Study methods

Diseases were classified according to the International Classification of Diseases, Tenth Revision. Tuberculosis case data for the global population, China, Japan, and South Korea were included, with sex categorized as male, female, and both sexes combined, covering the period from 1990 to 2023. The following age groups were selected: 15 to 19, 20 to 24, 25 to 29, 30 to 34, 35 to 39, 40 to 44, 45 to 49, 50 to 54, 55 to 59, 60 to 64, 65 to 69, 70 to 74, and 75 to 79 years. Incidence, prevalence, mortality, and DALYs data stratified by sex and age group for China, Japan, and South Korea in 2023 were summarized, and the estimated annual percentage change (EAPC) with its 95% confidence interval (CI) for the age-standardized incidence rate (ASIR), age-standardized prevalence rate (ASPR), age-standardized mortality rate (ASMR), and age-standardized DALYs rate (ASDR) from 1990 to 2023 were calculated. The age-standardized rate was calculated using the following formula^[13]^:


$$\begin{array}{c} Age\text{-}Standardized\;\;\;Rate=\frac{\overset{n}{\underset{i=1}{\sum }}r_{i}\times w_{i}}{\overset{n}{\underset{i=1}{\sum }}w_{i}}\times 1,00,000 \end{array},$$ (1)

where *r*_*i*_ denotes the estimated rate (e.g., mortality rate, incidence rate, or DALYs rate) for the *i*th age group, *w*_*i*_ is the weight of the *i*th age group in the GBD world standard population, and n is the total number of age groups.

The EAPC was calculated by fitting a linear regression model to assess temporal trends in annual health indicators. An EAPC value <0 with a 95% CI that excludes 0 indicates a statistically significant average annual decline in the indicator; conversely, an EAPC value greater than 0 with a 95% CI that excludes 0 indicates a statistically significant average annual increase.

For the analysis of attributable risk factors, negative binomial regression was used to estimate relative risks, from which the population attributable fraction for each risk factor was calculated to assess the attributable disease burden of smoking, high alcohol use, and high fasting plasma glucose on tuberculosis deaths and DALYs in the 3 countries. The population attributable fraction for risk factor *i* in region *k* was calculated using the following formula^[14]^:


$$\begin{array}{c} PAF_{ijk}=\frac{\overset{x_{max}}{\underset{x_{min}}{\int }}RR_{ij}\left(x\right)\;P_{ik}\left(x\right)\;dx-\overset{x_{max}}{\underset{x_{min}}{\int }}RR_{ij}\left(x\right)\;\overset{*}{\underset{ij}{P}}\left(x\right)\;dx}{\overset{x_{max}}{\underset{x_{min}}{\int }}RR_{ij}\left(x\right)\;P_{ik}\left(x\right)\;dx} \end{array}.$$ (2)

The BAPC model, grounded in a Bayesian statistical framework, comprehensively accounts for age effects, period effects, and cohort effects to analyze the dynamic changes in disease burden and project future trends. In this study, the BAPC model was applied to project the ASIR, ASPR, ASMR, and ASDR of tuberculosis in China, Japan, and South Korea from 2023 to 2035. The BAPC framework decomposes the log-transformed event rate into 3 additive components – age, period, and cohort effects – assumed to evolve smoothly over time according to second-order random walks. The model further assumes that observed counts follow an overdispersed Poisson distribution and that historical trends will continue without abrupt structural change during the projection horizon. Model estimation was implemented using the Integrated Nested Laplace Approximation method, which provides accurate posterior approximations without the need for computationally intensive Markov Chain Monte Carlo sampling. The prior distribution for the age effect was specified as follows^[15]^:


$$\;\;\;\begin{array}{c} p\left(\alpha |k_{\alpha }\right)\propto exp\left(-\frac{k_{\alpha }}{2}\underset{i=3}{\overset{t}{\sum }}\;{\left(\alpha_{i}-2\alpha_{i-1}+\alpha_{i-2}\right)}^{2}\right) \end{array}.$$ (3)

The log model for the event count in age group α during period t over the projection horizon is specified as follows^[16]^:


$$\begin{array}{c} log\left(Y_{\alpha ,\;p+t}\right)=\mu +\alpha +\beta +\gamma +\delta_{\alpha ,\;p+t}, \end{array}$$ (4)

where δ_α,*p*+*t*_ ∼ N(0, *k*^−1^) is an independent random effects term used to account for overdispersion. The prior distribution for the period effect was specified as follows^[17]^:


$$\begin{array}{c} \beta_{p+1}|\beta_{1},\ldots ,\beta_{p}\sim N\left(\beta_{p}+\left(\beta_{p}-\beta_{p-1}\right),\;\frac{1}{k_{\beta }}\right) \end{array}.$$ (5)

### 2.3. Statistical analysis

Data management was performed using Excel 2023 and the tidyverse and reshape2 packages in R 4.4.1. EAPC analysis was conducted using Joinpoint software (version 4.9.1.0). BAPC analysis was carried out using the BAPC package in conjunction with the integrated nested laplace approximation package. Data visualization was achieved using the GBDR, ggpubr, and ggplot2 packages.

## 3. Results

### 3.1. Disease burden

**Figure 1.**
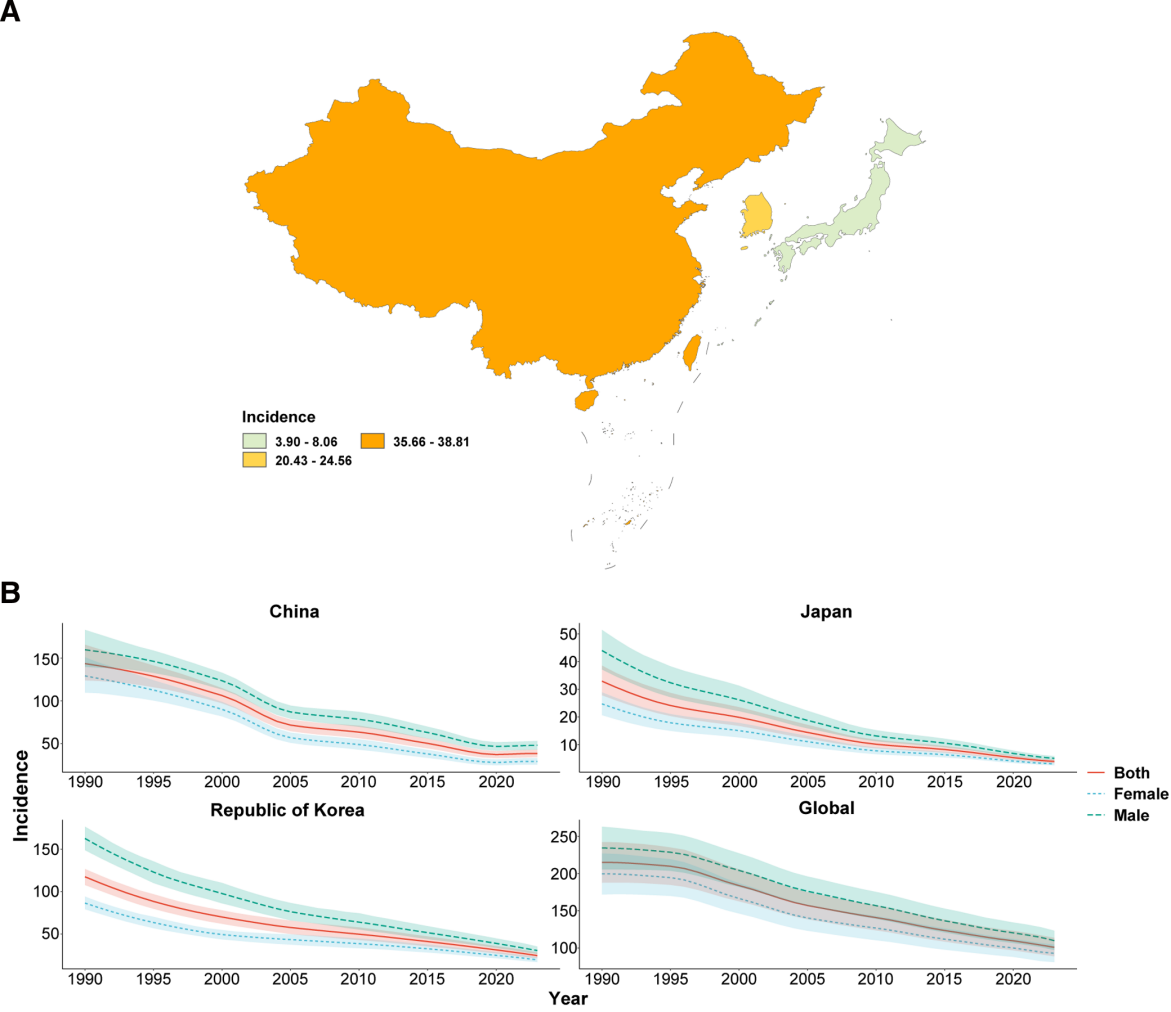
ASIR of tuberculosis. (A) Age-standardized incidence rate (ASIR) of tuberculosis in China, Japan, South Korea, and globally in 2023; (B) temporal trends in ASIR from 1990 to 2023 stratified by sex. ASIR = age-standardized incidence rate.

**Figure 2.**
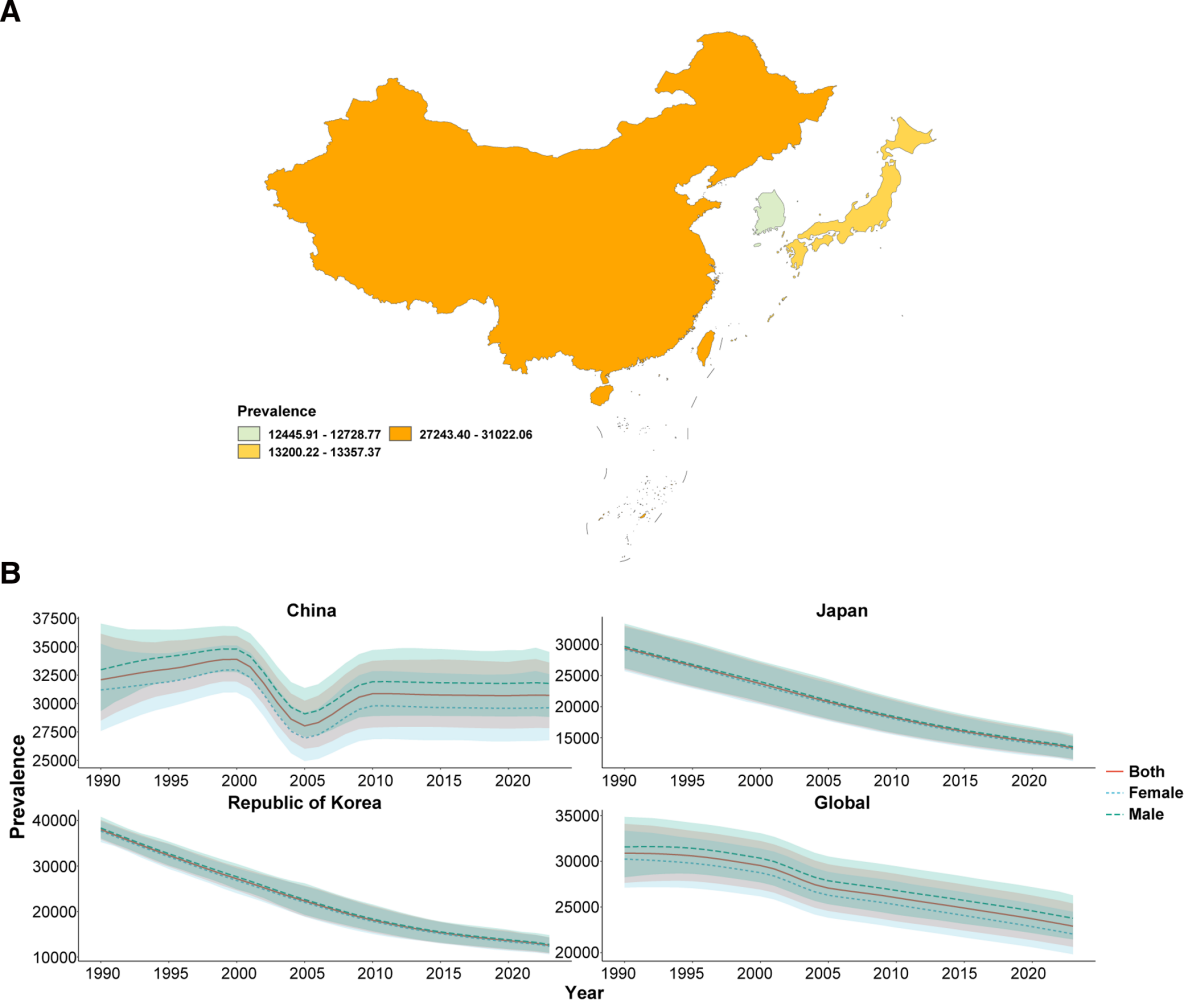
ASPR of tuberculosis. (A) Age-standardized prevalence rate (ASPR) of tuberculosis in China, Japan, South Korea, and globally in 2023; (B) yemporal trends in ASPR from 1990 to 2023 stratified by sex. ASPR = age-standardized prevalence rate.

**Figure 3.**
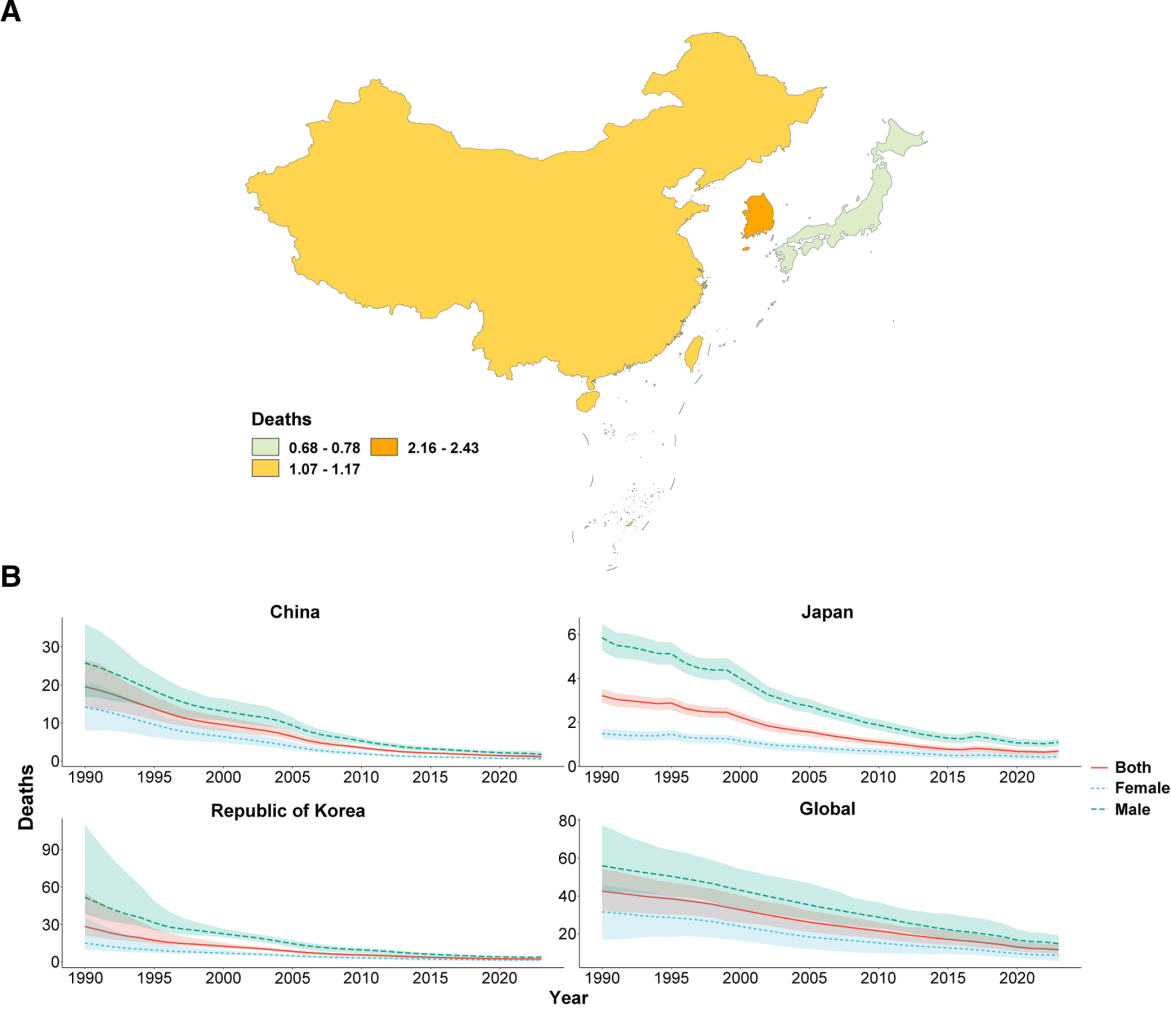
ASMR of tuberculosis. (A) Age-standardized mortality rate (ASMR) of tuberculosis in China, Japan, South Korea, and globally in 2023; (B) temporal trends in ASMR from 1990 to 2023 stratified by sex. ASMR = age-standardized mortality rate.

**Figure 4.**
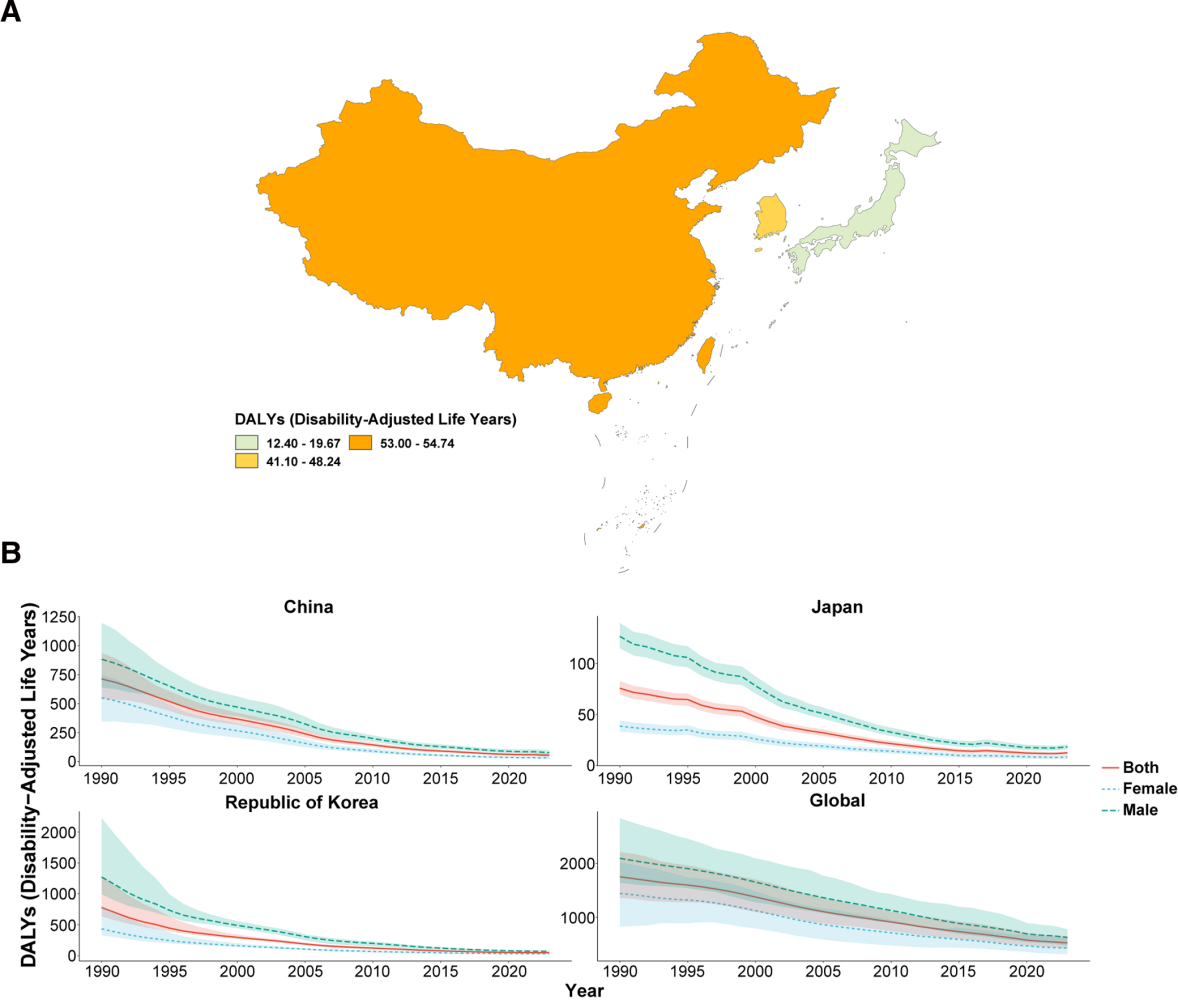
ASDR of tuberculosis. (A) Age-standardized DALYs rate (ASDR) of tuberculosis in China, Japan, South Korea, and globally in 2023; (B) temporal trends in ASDR from 1990 to 2023 stratified by sex. ASDR = age-standardized disability-adjusted life years rate.

In 2023, the ASIR, ASPR, ASMR, and ASDR of TB in China, Japan, and South Korea were all below the global average, with Japan consistently showing the lowest burden and China the highest ASIR and ASPR (Figs. 1A–4A; Tables 1–4). From 1990 to 2023, all 4 age-standardized indicators declined significantly in the 3 countries, with EAPCs whose 95% CIs excluded 0, indicating statistically significant downward trends.

**Table 1:** Age-standardized incidence rate (ASIR) of tuberculosis and its temporal trends from 1990 to 2023.

Country	ASIR per 1,00,000 population (95% UI)		EAPCs (95% CI)
	1990	2023	
China	143.66 (123.99, 165.71)	38.43 (34.01, 42.94)	−4.49 (−4.67, −4.30)
Japan	32.92 (27.86, 38.54)	3.94 (3.33, 4.60)	−5.88 (−6.10, −5.67)
South Korea	117.49 (107.81, 126.61)	24.56 (21.54, 28.17)	−4.11 (−4.26, −3.95)
Global	215.11 (187.80, 242.44)	100.81 (88.68, 113.56)	−2.45 (−2.53, −2.38)

ASIR = age-standardized incidence rate, CI = confidence interval, EAPC = estimated annual percentage change, UI = uncertainty interval.

**Table 2:** Age-standardized prevalence rate (ASPR) of tuberculosis and its temporal trends from 1990 to 2023.

Country	ASPR 1,00,000 population (95% UI)		EAPCs (95% CI)
	1990	2023	
China	32,089.04 (28,495.54, 36,135.56)	30,714.91 (27,838.23, 33,598.70)	−0.27 (−0.42, −0.11)
Japan	29,426.77 (25,970.48, 33,038.19)	13,357.37 (11,456.29, 15,321.20)	−2.44 (−2.47, −2.41)
South Korea	38,000.44 (35,814.83, 40,085.46)	12,571.63 (10,806.28, 14,330.15)	−3.46 (−3.55, −3.37)
Global	30,890.34 (27,651.32, 34,092.88)	22,902.75 (20,645.35, 25,374.13)	−0.99 (−1.03, −0.94)

ASPR = age-standardized prevalence rate, CI = confidence interval, EAPC = estimated annual percentage change, UI = uncertainty interval.

**Table 3:** Age-standardized mortality rate (ASMR) of tuberculosis and its temporal trends from 1990 to 2023.

Country	ASMR per 1,00,000 population (95% UI)		EAPCs (95% CI)
	1990	2023	
China	19.50 (14.09, 26.34)	1.17 (0.86, 1.64)	−8.61 (−8.86, −8.36)
Japan	3.21 (2.89, 3.52)	0.68 (0.55, 0.79)	−5.37 (−5.62, −5.11)
South Korea	28.25 (21.48, 55.06)	2.41 (1.88, 2.80)	−7.47 (−7.63, −7.31)
Global	42.59 (32.43, 54.20)	11.64 (9.23, 14.41)	−4.00 (−4.14, −3.85)

ASMR = age-standardized mortality rate, CI = confidence interval, EAPC = estimated annual percentage change, UI = uncertainty interval.

**Table 4:** Age-standardized DALYs rate (ASDR) of tuberculosis and its temporal trends from 1990 to 2023.

Country	ASDR per 1,00,000 population (95% UI)		EAPCs (95% CI)
	1990	2023	
China	713.39 (545.16, 934.90)	54.20 (42.90, 70.09)	−3.79 (−3.92, −3.66)
Japan	75.95 (69.65, 82.82)	12.53 (10.67, 14.18)	−6.21 (−6.48, −5.94)
South Korea	777.30 (634.37, 1275.50)	48.24 (41.04, 55.12)	−8.31 (−8.48, −8.14)
Global	1752.37 (1360.16, 2210.16)	528.09 (425.91, 654.03)	−3.79 (−3.92, −3.66)

ASDR = age-standardized disability-adjusted life years (DALYs) rate, CI = confidence interval, EAPC = estimated annual percentage change, UI = uncertainty interval.

The rate of decline differed markedly across indicators and countries. For ASIR and ASMR, all 3 countries outpaced the global decline (global EAPC = −2.45 and −4.00, respectively), with China showing the steepest mortality reduction (EAPC = −8.61, 95% CI: −8.86, −8.36; Table 3). For ASPR, China’s decline (EAPC = −0.27) was substantially slower than that of Japan (−2.44) and South Korea (−3.46), suggesting a persistently heavy prevalent TB burden (Table 2). For ASDR, South Korea showed the greatest reduction (EAPC = −8.31), whereas China’s decline matched the global average (both EAPC = −3.79; Table 4). Across all indicators and throughout the study period, males consistently bore a higher burden than females in all 3 countries (Figs. 1B–4B).

### 3.2. Age and sex differences

**Figure 5.**
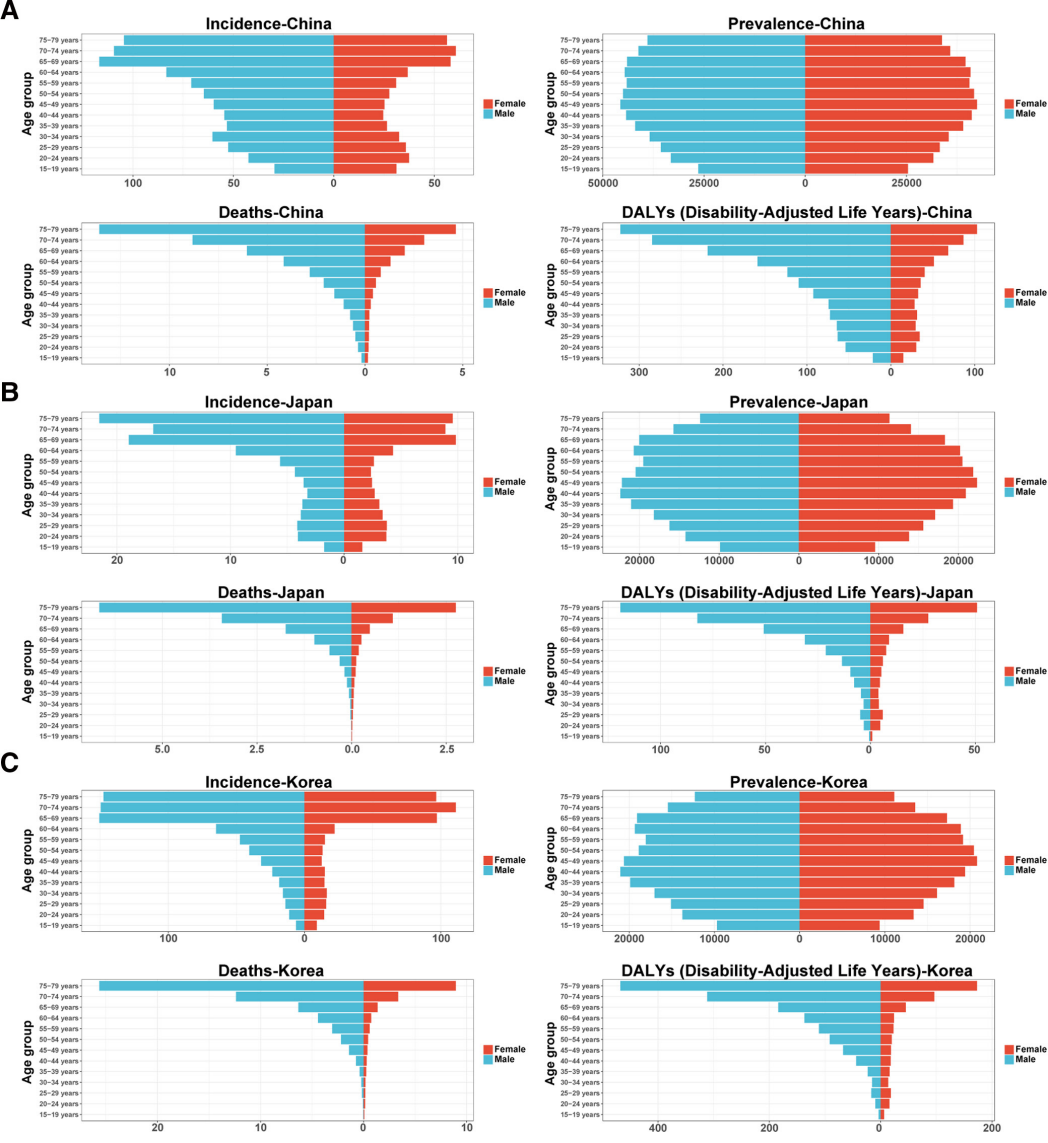
Age and sex distribution of tuberculosis burden in 2023. (A) China; (B) Japan; (C) South Korea. Incidence, prevalence, mortality, and DALYs are presented by age group and sex. DALY = disability-adjusted life year.

In 2023, the incidence, prevalence, mortality, and DALYs of tuberculosis increased with age across all 3 countries, with the heaviest burden observed in elderly populations, and males consistently bearing a greater burden than females across age groups (Fig. 5A–C). In Chia, sex differences in incidence became apparent from age 25 onward and widened progressively with age. Prevalence peaked in the 45 to 49-year age group. The sex gap in mortality and DALYs widened markedly after age 45, reaching its maximum in the 75 to 79-year group. Notably, females had incidence rates comparable to or slightly higher than males in the 15 to 24-year age group (Fig. 5A). In Japan, incidence, mortality, and DALYs rose sharply among older adults (≥65 years), with male rates peaking in the 75 to 79-year group. Female prevalence was slightly higher than or comparable to male prevalence in the 45 to 59-year age group (Fig. 5B). In South Korea, the disease burden was particularly concentrated in elderly populations, with male incidence in the 65 to 69-year group reaching 150.57 per 1,00,000. Prevalence remained elevated among the working-age population (30–60 years). The DALYs rate among males aged 75 to 79 years (467.39 per 1,00,000) was approximately 2.7 times that of females in the same age group (172.99 per 1,00,000), representing the most pronounced sex disparity among the 3 countries (Fig. 5C).

### 3.3. Attributable risk factors

**Figure 6.**
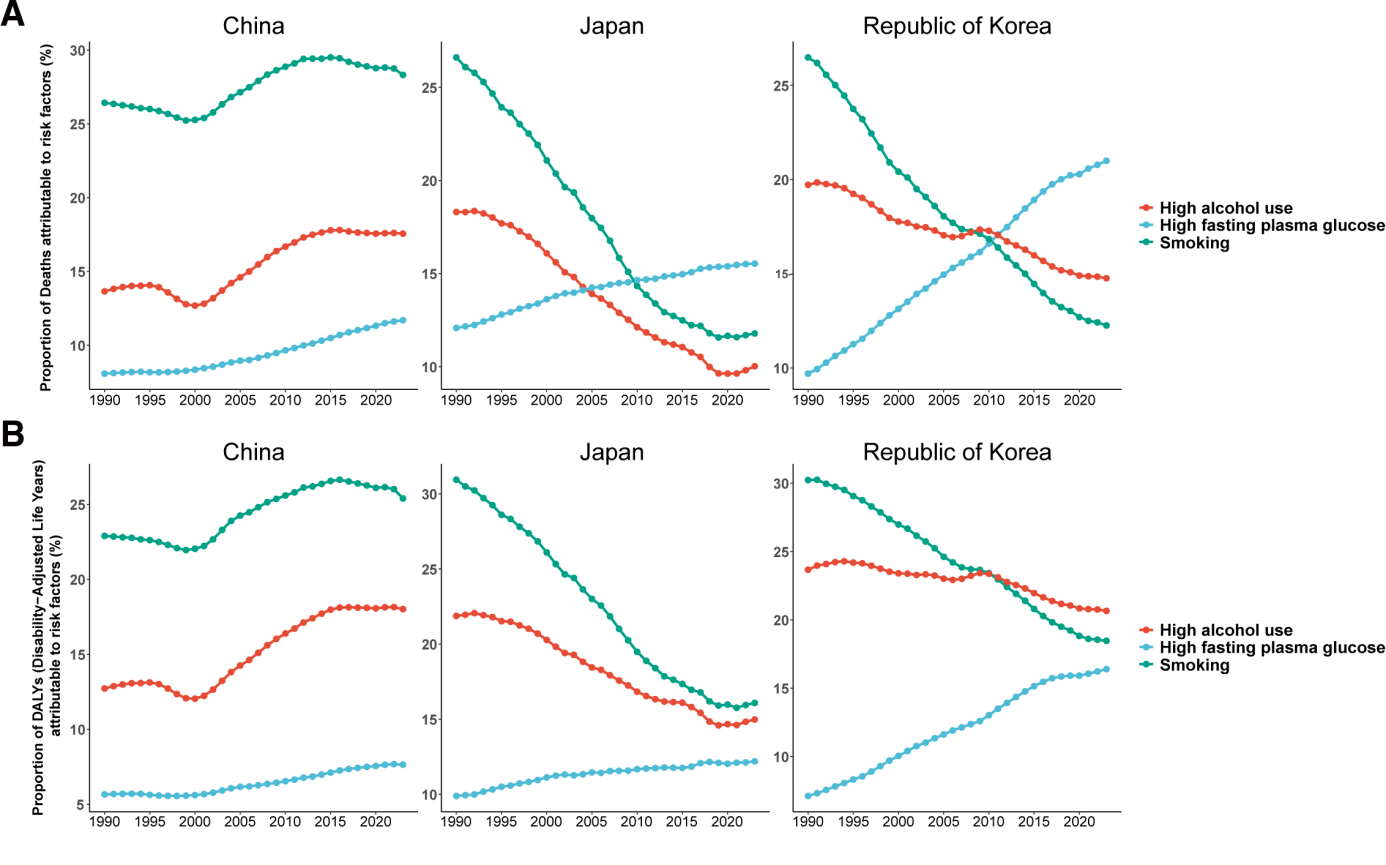
Trends in attributable risk factors for tuberculosis from 1990 to 2023. (A) Proportion of deaths attributable to risk factors; (B) proportion of DALYs attributable to risk factors. Risk factors include smoking, high fasting plasma glucose, and alcohol use. DALY = disability-adjusted life year.

From 1990 to 2023, smoking, high alcohol use, and high fasting plasma glucose were the primary risk factors attributable to tuberculosis deaths and DALYs in all 3 countries (Fig. 6A, B). Smoking remained the leading attributable factor across all 3 countries; however, its proportional contribution declined substantially in Japan and South Korea, whereas in China it initially rose before declining gradually, remaining the highest among the 3 countries in 2023 (approximately 25–28%). The attributable proportion of high alcohol use decreased in Japan and South Korea while remaining relatively stable in China. Notably, the attributable proportion of high fasting plasma glucose increased continuously in all 3 countries, most markedly in South Korea, where it surpassed smoking by the end of the study period to become the leading attributable risk factor for both tuberculosis deaths and DALYs, highlighting the growing importance of metabolic risk factors in tuberculosis control.

### 3.4. Predictions of disease burden trends

**Figure 7.**
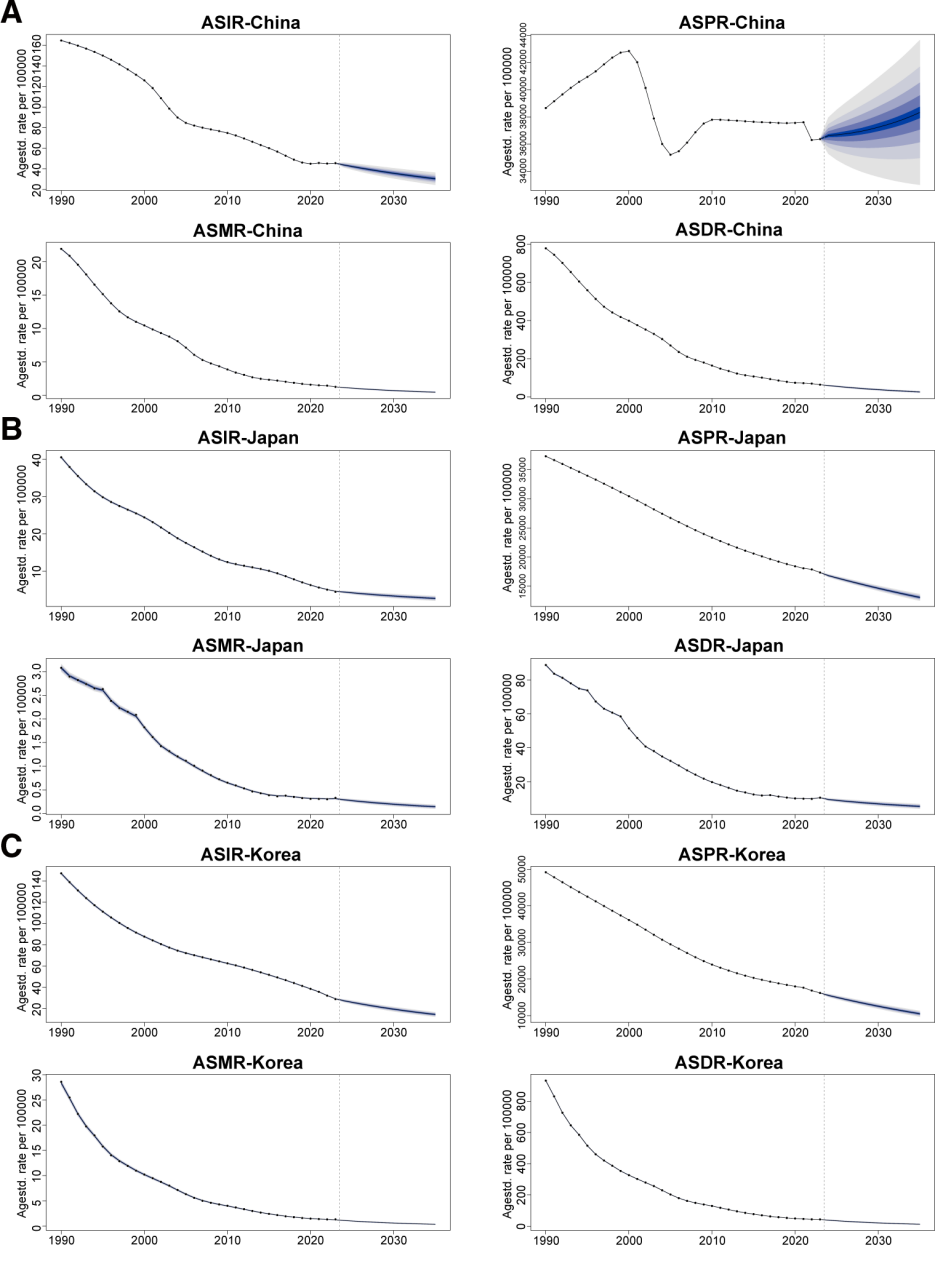
Predicted trends of tuberculosis burden from 2023 to 2035. (A) China; (B) Japan; (C) South Korea. Projections of ASIR, ASPR, ASMR, and ASDR are shown based on the BAPC model. The shaded areas represent uncertainty intervals (50–95%). ASDR = age-standardized disability-adjusted life year rate, ASIR = age-standardized incidence rate, ASMR = age-standardized mortality rate, ASPR = age-standardized prevalence rate, BAPC = Bayesian age-period-cohort.

According to BAPC model projections, the overall tuberculosis burden in all 3 countries is expected to continue declining from 2023 to 2035 (Fig. 7). By 2035, China’s projected ASIR, ASMR, and ASDR are approximately 30.54, 0.45, and 25.62 per 1,00,000, respectively; however, ASPR is predicted to fluctuate with a slight initial increase before stabilizing (approximately 38,323 per 1,00,000), indicating persistent challenges in controlling the prevalent tuberculosis burden. In Japan, all 4 indicators are projected to decline steadily, with ASIR and ASMR expected to reach approximately 2.72 and 0.14 per 1,00,000, respectively, by 2035. In South Korea, all 4 indicators are likewise projected to continue declining, with ASIR and ASMR reaching approximately 14.52 and 0.35 per 1,00,000, respectively, by 2035, reflecting the sustained effectiveness of current control measures.

## 4. Discussion

Despite global progress, TB control trajectories vary widely across East Asia, and a long-term comparative analysis of China, Japan, and South Korea with future projections has been lacking. Using the GBD 2023 database, this study systematically assessed TB incidence, prevalence, mortality, and DALYs from 1990 to 2023 and projected trends to 2035. These findings collectively suggest that the next phase of TB control in East Asia must move beyond traditional case-finding toward the integrated management of aging populations and metabolic comorbidities.

The rates of decline in ASIR across all 3 countries were significantly faster than the global average, which is closely associated with their relatively well-developed healthcare systems and proactive TB control policies. Nevertheless, the absolute gap in incidence levels among the 3 countries remains substantial: The 10-fold gap in ASIR between Japan and China reflects longstanding structural disparities in healthcare access, population density, and screening coverage,^[18]^ who reported similar longstanding structural disparities between high-burden and low-burden East Asian countries in economic development, population density, healthcare resource accessibility, and early screening coverage. Of particular note, the decline in China’s ASPR (EAPC = −0.27) lags far behind that of Japan (EAPC = −2.44) and South Korea (EAPC = −3.46), suggesting that even as new cases continue to decrease, ongoing transmission from previously infected individuals remains a serious concern. Large-scale screening for latent TB infection and preventive treatment may therefore represent important future priorities.^[19]^

The improvement in mortality burden is equally noteworthy. The ASMR across all 3 countries has already fallen well below the global average, with China recording the greatest decline (EAPC = −8.61), attributable to the sustained advancement of the National TB Control Programme and marked improvements in access to anti-TB medications.^[20]^ Nevertheless, South Korea’s 2023 ASMR (2.41 per 1,00,000) remains higher than those of China (1.17 per 1,00,000) and Japan (0.68 per 1,00,000), which may be related to South Korea’s more advanced population aging, the heavier comorbidity burden among elderly patients, and the issue of multidrug-resistant TB.^[21]^ BAPC model projections indicate that the ASMR in all 3 countries will continue to decline, with the potential to reach extremely low levels by 2035; however, achieving the World Health Organization End TB Strategy targets will still require sustained efforts in treatment quality, drug resistance surveillance, and cross-sectoral collaboration.

Regarding age and sex distribution, the TB burden in all 3 countries increases markedly with age, with the elderly representing the highest-risk group, and males consistently bearing a greater burden than females across all age groups. Immunosenescence in older adults, combined with a higher likelihood of comorbidities such as diabetes and chronic pulmonary disease, largely explains their heightened susceptibility to TB infection. This mechanism is consistent with the findings of Teo et al,^[5]^ who emphasized that age-related immunosenescence, together with common comorbidities such as diabetes and chronic respiratory diseases, significantly increases the vulnerability of older adults to TB reactivation and progression in rapidly aging East Asian populations. The higher burden among males is closely linked to behavioral factors such as elevated smoking rates, higher alcohol consumption, and delays in seeking medical care. This is supported by Yang et al,^[8]^ demonstrating that men exhibit higher rates of smoking and alcohol use, leading to greater attributable TB risk. Notably, TB incidence rates among females aged 15 to 24 in China and Japan approach or slightly exceed those of their male counterparts, a phenomenon that may be related to the distinctive immune status, hormonal fluctuations, or specific social exposure patterns of young women and warrants further investigation.^[4,22]^ As the aging process continues to accelerate across East Asia, proactive screening and comprehensive management strategies targeting elderly populations should be prioritized.

The dynamic changes in risk factor profiles also reveal trends deserving attention. Smoking has long been the dominant attributable factor in all 3 countries, but its proportional contribution has shown a marked declining trend in Japan and South Korea, consistent with the progress of tobacco control policies and the decline in population smoking rates in both countries.^[23]^ In stark contrast, the attributable contribution of high fasting plasma glucose increased continuously across the 3 countries, most prominently in South Korea, where it surpassed smoking to become the leading attributable risk factor for TB deaths and DALYs by the end of the study period. This finding is consistent with previous evidence indicating that diabetes substantially increases susceptibility to TB, with an approximately 2- to 3-fold higher risk compared with nondiabetic individuals, potentially due to impaired cellular immune responses.^[24]^ As diabetes prevalence continues to rise across East Asia, it is now imperative to integrate TB screening into routine diabetes management and to establish a coordinated prevention and control framework addressing both conditions. In addition, the attributable proportion of smoking in China remains the highest among the 3 countries (approximately 25–28%), reinforcing the practical significance of intensifying tobacco control interventions for TB prevention in China.^[25]^

Regarding the projection findings, the overall TB burden in all 3 countries is expected to continue improving before 2035. However, China’s ASPR is projected to experience a slight initial increase before stabilizing, an anomalous fluctuation likely linked to China’s enormous pool of latent TB infections and the increased risk of endogenous reactivation against a backdrop of population aging,^[26]^ revealing the deep-seated challenges in controlling the prevalent burden. While continuing to advance standardized treatment and management, there is an urgent need to systematically strengthen interventions targeting latent infections in high-risk populations in order to fundamentally reduce the reservoir of infectious sources and drive a meaningful decline in prevalence.

BAPC projections should be interpreted as conditional on the continuation of recent trends; the widening credible intervals toward 2035 underscore that point estimates for that year should be read alongside their uncertainty bounds rather than as exact forecasts. Projections should therefore be interpreted as conditional on the continuation of recent dynamics. The 95% uncertainty intervals (UIs) reported alongside our estimates merit careful interpretation. Where UIs across countries do not overlap – such as the contrast between Japan’s and China’s ASIR in 2023 – between-country differences can be considered robust; where UIs partially overlap, as for some risk-factor population attributable fractions, apparent differences should be interpreted as suggestive rather than definitive. For BAPC projections, the credible intervals widen progressively from 2024 to 2035, reflecting compounding extrapolation uncertainty; point estimates for 2035 should therefore be read together with their intervals rather than as exact forecasts.

This study has several limitations. GBD estimates are modeled rather than directly measured, relying on heterogeneous primary data and covariate-based imputation where data are sparse. Variation in input quality across countries and years introduces uncertainty that may affect cross-country comparability. Second, the analysis was restricted to 3 GBD-quantified risks: smoking, high alcohol use, and high fasting plasma glucose. Key determinants such as undernutrition and air pollution were excluded due to GBD framework limitations. Consequently, the total attributable burden – particularly among the elderly and disadvantaged – may be underestimated. The BAPC model’s projections are based on linear extrapolation of historical trends and cannot fully account for uncertainties such as future policy changes, the dissemination of novel diagnostic and therapeutic technologies, or unexpected public health emergencies; the projection results therefore warrant cautious interpretation in light of actual circumstances.

## 5. Conclusion

From 1990 to 2023, the tuberculosis disease burden in China, Japan, and South Korea declined continuously, although notable differences exist in the pace of progress. Accelerating population aging and the rising attributable contribution of high fasting plasma glucose represent shared challenges across all 3 countries. Projections indicate that the burden will continue to decline, though China’s prevalence rate remains at risk of fluctuation. Going forward, it will be essential to further strengthen latent infection interventions, high-risk population screening, and comprehensive management of metabolic risk factors in order to accelerate the achievement of regional TB control targets.

## Acknowledgments

The authors would like to express their sincere gratitude to the Global Burden of Disease Study 2023 collaborators and the Institute for Health Metrics and Evaluation (IHME) at the University of Washington for providing open access to the GBD 2023 database. The findings and conclusions presented in this paper are solely those of the authors and do not necessarily represent the official views or positions of the IHME or its funding organizations.

## Author contributions

**Conceptualization:** Huan Yao.

**Data curation:** Huan Yao, Yujie Qian.

**Formal analysis:** Huan Yao, Zhi Xu.

**Investigation:** Huan Yao, Zhi Xu.

**Methodology:** Huan Yao, Zhi Xu.

**Software:** Yujie Qian, Zhi Xu.

**Supervision:** Yujie Qian.

**Writing – original draft:** Huan Yao, Zhi Xu.

**Writing – review & editing:** Yujie Qian.

## References

[1] Sahu S Ditiu L Ahmed R. Overcoming the global tuberculosis crisis with urgent country-level political and financial action. Lancet Infect Dis. 2025;25:14–6. 39577455 10.1016/S1473-3099(24)00748-5

[2] Lee H Kim J Choi H. Review on Global Burden of Tuberculosis in 2022. Jugan Geongang Gwa Jilbyeong. 2024;17:438–51. 41333196 10.56786/PHWR.2024.17.11.2 PMC12480281

[3] Garcia-Bereguiain MA Rodriguez-Pazmiño AS Franco-Sotomayor G Orlando SA González M Ugarte-Gil C. “The end TB strategy” pathway in South America: out of tck for 2025 milestones and 2035 eradication. Lancet Reg Health Am. 2025;44:101045. 40124589 10.1016/j.lana.2025.101045 PMC11928972

[4] Li N Wei W Lao Y. Long-term trends and future projections of the burden of tuberculosis among children and adolescents in China. PLoS One. 2025;20:e0328255. 40674435 10.1371/journal.pone.0328255 PMC12270101

[5] Teo AKJ Rahevar K Morishita F. Tuberculosis in older adults: case studies from four countries with rapidly ageing populations in the western pacific region. BMC Public Health. 2023;23:370. 36810018 10.1186/s12889-023-15197-7 PMC9942033

[6] Lee SW Jeon D Choi H. The impact of population ageing on tuberculosis incidence, mortality, and case fatality in South Korea: a nationwide retrospective study. ERJ Open Res. 2025;11:00191–2025. 41220818 10.1183/23120541.00191-2025 PMC12598592

[7] Kou Y Wang S Shi J. Analysis of the disease burden and attributable risk factors of tuberculosis in G20 countries from 1990 to 2021. BMC Infect Dis. 2025;25:1503. 41194007 10.1186/s12879-025-11843-0 PMC12587730

[8] Yang H Ruan X Li W Xiong J Zheng Y. Global, regional, and national burden of tuberculosis and attributable risk factors for 204 countries and territories, 1990-2021: a systematic analysis for the Global Burden of Diseases 2021 study. BMC Public Health. 2024;24:3111. 39529028 10.1186/s12889-024-20664-w PMC11552311

[9] Bian Q Zhang Y Xue C. Global and regional estimates of tuberculosis burden attributed to high fasting plasma glucose from 1990 to 2019: emphasis on earlier glycemic control. BMC Public Health. 2024;24:782. 38481192 10.1186/s12889-024-18260-z PMC10935816

[10] Yayan J Rasche K. Tuberculosis in older adults (≥ 65 years): global trends, sex differences, and regional variations, 2000-2023. BMC Geriatr. 2026;26:133. 41519733 10.1186/s12877-025-06934-1 PMC12857025

[11] Qin W Liu X Nong J. Global, regional, national burden and trends of unintentional injuries from 1990 to 2021 and projections to 2035: a systematic analysis of the Global Burden of Disease study 2021. Front Public Health. 2025;13:1653491. 40969640 10.3389/fpubh.2025.1653491 PMC12442766

[12] GBD 2023 Causes of Death Collaborators. Global burden of 292 causes of death in 204 countries and territories and 660 subnational locations, 1990–2023: a systematic analysis for the Global Burden of Disease Study 2023. Lancet. 2025;406:1811–72. 41092928 10.1016/S0140-6736(25)01917-8 PMC12535838

[13] Luo J Zhang Y Luo Z. Assessing the global burden of Type 2 diabetes in women of reproductive age. PLoS One. 2025;20:e0322787. 40658669 10.1371/journal.pone.0322787 PMC12258576

[14] Ezzati M Hoorn SV Rodgers A Lopez AD Mathers CD Murray CJ; Comparative Risk Assessment Collaborating Group. Estimates of global and regional potential health gains from reducing multiple major risk factors. Lancet. 2003;362:271–80. 12892956 10.1016/s0140-6736(03)13968-2

[15] Liu Q Wang H Chen Z. Global, regional, and national epidemiology of nasopharyngeal carcinoma in middle-aged and elderly patients from 1990 to 2021. Ageing Res Rev. 2025;104:102613. 39626854 10.1016/j.arr.2024.102613

[16] Li C Xu K Du A Fu N Xu Z Chang Q. Global, regional and national epidemiology of myocarditis: health inequalities, risk factors and forecasted burden based on the Global Burden of Disease Study 2021. Heart. 2025;111:867–76. 40246334 10.1136/heartjnl-2024-325523 PMC12421102

[17] Liu Z Xu K Jiang Y. Global trend of aetiology-based primary liver cancer incidence from 1990 to 2030: a modelling study. Int J Epidemiol. 2021;50:128–42. 33349860 10.1093/ije/dyaa196

[18] Lu ZQ Feng SC Feng M Shen J. Analysis of the trends and predictions of tuberculosis burden in China from 1990 to 2021 based on the GBD database. Front Public Health. 2025;13:1626232. 40959629 10.3389/fpubh.2025.1626232 PMC12434080

[19] Jiang QQ Zhang XY Li SX. Latent tuberculosis infection in China, 1990-2050: GBD-informed projections for age-targeted screening. Front Public Health. 2025;13:1691385. 41561873 10.3389/fpubh.2025.1691385 PMC12813108

[20] Zhang X Guo M Song X Abdalla AE Wang G Xie L. Comprehensive analysis of tuberculosis burden trends and attributable risk factors in the BRICS countries from 1990 to 2021, with forecasts for the next 15 years. Int J Surg. 2025;111:6050–62. 40540545 10.1097/JS9.0000000000002720 PMC12430850

[21] Liu J Zhou Y Guan J. Global burden of tuberculosis among adults aged 60 years and older, 1990-2021: findings from the global burden of disease study 2021. Int J Infect Dis. 2025;158:107966. 40581250 10.1016/j.ijid.2025.107966

[22] Min J Park JS Kim HW. Differential effects of sex on tuberculosis location and severity across the lifespan. Sci Rep. 2023;13:6023. 37055508 10.1038/s41598-023-33245-5 PMC10102118

[23] Zhao G Wu Y Song C. Global, regional, and national burden of tuberculosis due to smoking, 1990-2021: analysis for the Global Burden of Disease study. Front Immunol. 2025;16:1624090. 40787439 10.3389/fimmu.2025.1624090 PMC12331698

[24] Ssekamatte P Sande OJ van Crevel R Biraro IA. Immunologic, metabolic and genetic impact of diabetes on tuberculosis susceptibility. Front Immunol. 2023;14:1122255. 36756113 10.3389/fimmu.2023.1122255 PMC9899803

[25] Boadu AA Yeboah-Manu M Osei-Wusu S Yeboah-Manu D. Tuberculosis and diabetes mellitus: the complexity of the comorbid interactions. Int J Infect Dis. 2024;146:107140. 38885832 10.1016/j.ijid.2024.107140

[26] Zhu Z Shen W Hu J. Risk factors for latent tuberculosis infection clustering among the elderly: a population-based cross-sectional study in Eastern China. BMC Infect Dis. 2025;25:368. 40098074 10.1186/s12879-025-10743-7 PMC11912638

